# The Pandemic Is Not Occurring in a Vacuum: The Impact of COVID-19 and Other Disasters on Workforce Mental Health in Australia

**DOI:** 10.1017/dmp.2021.238

**Published:** 2021-07-23

**Authors:** Tegan Podubinski, Kristen M. Glenister

**Affiliations:** Department of Rural Health, University of Melbourne, Wangaratta, Victoria, Australia

**Keywords:** anxiety, COVID-19, depression, disasters, mental health, psychological distress, stress

## Abstract

**Objective:**

Prior to coronavirus disease (COVID-19), many Australians experienced extreme bushfires, droughts, and floods. A history of experiencing these events might be a risk factor for increased psychological distress during COVID-19. This study aimed to provide insight into the mental health of Australian workers during the initial COVID-19 outbreak, with an additional focus on whether previous disaster exposure and impact from that disaster is a risk factor for increased psychological distress.

**Methods:**

A snowball recruitment strategy was used. Participants (n = 596) completed an online survey, which included the Depression Anxiety Stress Scales-21, and questions related to mental health and disaster exposure.

**Results:**

Overall, 19.2%, 13.4%, and 16.8% of participants were experiencing moderate to extremely severe depression, anxiety, and stress symptoms, respectively. Multiple regression found that higher depression, anxiety, and stress symptoms were associated with a pre-existing mental health diagnosis; only higher stress symptoms were associated with having experienced a disaster, with impact, in addition to COVID-19.

**Conclusions:**

People who have experienced impact from an additional disaster might need additional support to protect their mental health during COVID-19. A focus on the cumulative mental health impacts of multiple disasters and the implications for organizational communities where recovery work is undertaken, such as schools and workplaces, is needed.

## Introduction

Within Australia, the coronavirus disease (COVID-19) pandemic has caused unprecedented disruption to the way most people live, with widespread physical distancing, travel restrictions and border closures, work-from-home mandates, and lockdowns enforced. Previous respiratory endemics, such as severe acute respiratory syndrome coronavirus (SARS-CoV), influenza A/H1N1, and the Middle East Respiratory Syndrome (MERS) posed significant psychological consequences onto many population groups, including symptoms of anxiety, depression, and posttraumatic stress disorder.^1^ Given the level of upheaval associated with COVID-19, it is likely there will be both an acute and long-term impact on the mental health and well-being of Australian communities, although the scale and extent of this is only now starting to emerge.^2^ The impacts of COVID-19 might also be compounded by other recent disasters. For example, prior to the COVID-19 pandemic, devastating bushfires ravaged parts of the country, and entire communities were unable to recover before the nation’s focus shifted to the threat of COVID-19. The psychological health impacts of these bushfires are still emerging.^3^ Rural Australia was predominantly impacted by these events,^4^ and, in fact, it is disproportionally impacted by extreme climate-related events in general, with bushfires, droughts, and floods common.^5^ This situation is unlikely to improve as climate-related disasters become more frequent and severe.^5^ Unfortunately, the Australian COVID-19 literature has largely focused on COVID-19 in isolation from other disasters.

International studies focused on measuring psychological distress associated with the COVID-19 pandemic using the Depression Anxiety Stress Scales-21 (DASS-21)^6^ have consistently drawn attention to the high levels of depression, anxiety, and stress-related symptoms being experienced by the general population during COVID-19. Studies from China, Mexico, and Italy have shown that between 15.7–32.4%, 18.7–28.8%, and 8.1–27.2% of people have experienced moderate to severe depression, anxiety, and stress symptoms, respectively.^7–10^ Within Australia, 2 studies have used the DASS-21 to assess psychological distress within the general Australian population during the COVID-19 pandemic.^2,11^ One study, conducted from March 27 to April 7, 2020, found that 46.3%, 40.8%, and 38.7% of respondents were experiencing moderate to extremely severe symptoms of depression, anxiety, and stress, respectively. However, it should be noted that a significant proportion of the sample had a lived experience of a mental health diagnosis (70%), which likely contributed to the high rates of psychological distress.^2^ Rates of psychological distress more consistent with international research were found in a separate Australian study, conducted from April 1 to 4, 2020. In this study, 21–35% of the population demonstrated moderate to extremely severe depression, anxiety, and stress, with the reported rates being greater than the population norms.^11^ These studies highlight the significant psychological impacts of the COVID-19 pandemic on the general population; however, it is likely the risk of experiencing psychological distress varies, depending on a person’s characteristics and vulnerabilities.^12^

The 2 aforementioned Australian studies also give some insights into the factors that place a person at higher risk of experiencing psychological distress. While there was variability between the studies in the specific types of factors that were found to be important, in general, the factors could be condensed into 3 overarching categories: demographic variables (eg, being female, a student, a career or stay-at-home parent), personal and financial vulnerabilities (eg, having a chronic illness or a prior history of mental health diagnosis, experiencing financial stress, loneliness), and COVID-19-specific worries and experiences (eg, having a higher mortality risk or perceiving higher illness severity). Furthermore, a number of variables stand out as being of particular relevance to a risk of increased psychological distress: having a chronic illness or a prior history of mental health diagnosis, and experiencing financial stress.^2,11^ Similar categories of risk factors have also been shown in international samples.^8–10^ Of particular relevance to the Australian context is 1 study that found that a history of stressful situations (eg, dismissal, mourning) prior to COVID-19 was associated with higher levels of depression and anxiety.^10^ This finding emphasizes the need to consider the mental health impacts of COVID-19 in combination with other life stressors, such as other disasters.

Following a disaster, such as bushfires or floods, the majority of people will recover psychologically and most will not go on to develop a mental health disorder. However, many people will experience strong emotional and physical reactions.^12–15^ At times, the adverse psychological effects can be experienced for prolonged periods after the disaster occurs, particularly when there is severe trauma exposure or additional adverse life events in the aftermath of the event.^16^ While there is a large amount of literature on disasters and mental health,^12,13^ relatively few studies have examined psychological outcomes after multiple disasters. Within Australia, exposure to multiple (ie, 2 or more) natural disasters (eg, flood, cyclone, or earthquake) has been associated with an increased lifetime risk of panic disorder, while cumulative effects of repeated man-made disaster exposures (eg, a fire started by a cigarette or a bomb explosion) have been noted in terms of an increased lifetime risk of obsessive compulsive disorder.^17^ Research has also identified cumulative effects of repeated disaster exposure (both man-made and natural) in relation to the risk of suicide at the Australian population level.^18^ These studies are limited in that the focus on diagnosable psychiatric disorders and suicidality might not be indicative of subclinical levels of distress.^14,15^ Taken together, these 2 studies justify a deeper analysis of the cumulative psychological impact of prior disaster experience and COVID-19. Disaster experience should not only include exposure, but also impact; given the level of psychological distress a person experiences following a disaster is impacted by how people are affected by the disaster.^19,20^

This paper forms part of a larger program of research looking at the impact of COVID-19 on organizations and workforce and how organizations can be used to deliver appropriate interventions to improve employee preparedness, response, and recovery. Investigating this issue is important as workplaces are an essential part of most people’s social support systems and might offer a level of long-term protection from the psychological impacts of pandemics and disasters,^15,21,22^ especially if the capacity of organizations to provide appropriate support can be developed.^23,24^ Further, novel approaches to community intervention need to be explored,^12^ particularly in rural and regional Australia where appropriate treatment services are often limited or unavailable.^25^ While these are larger issues that will be considered in other manuscripts, this paper specifically focuses on the mental health of Australian workers during the COVID-19 outbreak. It was hypothesized that Australian workers would report levels of anxiety, depression, and stress above the population norms.^21^ It was also hypothesized that previous recent disaster exposure and impact from that disaster will be a risk factor for higher levels of depression, anxiety, and stress.

### COVID-19 Public Health Response: Contextual Information

Prior to the survey’s distribution, people living in Australia had experienced a series of public health responses that varied depending on geographical location. Restrictions had included border closures, directives to stay at home unless for necessary shopping, health care, exercise and work and study that can’t be done remotely, and the closing of non-essential services and many schools. At the time of the survey’s distribution, these restrictions were gradually being eased. However, by June 20, restrictions were being reinstated in the state of Victoria due to an apparent “second wave” of infection. By July 7, 2020, a second period of lockdown had been introduced for metropolitan Melbourne and neighboring Mitchell Shire.^26,27^ As of July 8, 8886 confirmed cases (56% acquired from overseas) and 106 deaths (predominantly in people ages 60 and greater) had occurred in Australia.^28^

## Method

### Recruitment

A link to the online survey was distributed via e-mail and social media posts, initially using the authors’ personal and professional networks with subsequent snowball recruitment. The survey was available in English only. Inclusion criteria comprised residing in Australia, being age 18 years and over, and having volunteered or been employed at any time during the period from December 2019 to July 2020. Data were collected from May 29 to July 8, 2020. A follow-up survey will be conducted when restrictions have been lifted. Study data were collected and managed using REDCap electronic data capture tools hosted at The University of Melbourne.

### Ethics Approval and Consent

The study was conducted in compliance with the Declaration of Helsinki, the National Statement on Ethical Conduct in Human Research (2007, updated 2018) and the University of Melbourne’s COVID-19 Central Human Ethics Committee (Approval number: 2056921.1). All participants provided electronic informed consent before participating. Participants could choose to enter a prize draw for 1 US $100 gift card and/or register their interest in a follow-up survey, by providing an email address. If participants declined to enter the prize draw or register their interest in a follow-up survey, their responses remained anonymous.

### Participants

In total, 616 records were created in REDCap. Responses received after July 8, 2020, were removed from the data set. Of the remaining records, 596 participants were included in the analysis.

### Measures

#### Demographics

Information was collected on each participant’s age, gender, postcode of residence, living arrangements (*by myself, with my partner, with pre-school-aged children, with school-aged children, with friends or flatmates, with other family, other*), diagnosis of COVID-19 (*yes, tested and confirmed, suspected but not tested, no, don’t know)*, COVID-19 diagnosis in another person in the house hold (*yes, tested and confirmed, suspected but not tested, no, don’t know*), industry of work,^29^ nature of work (*paid, volunteer*), employment type (*full time, part time, casual, not applicable, other*), work location (*home, normal workplace, home and normal workplace combination, not currently working or volunteering, other*), and changes to employment due to the pandemic (*temporarily stood down, role terminated, hours reduced, hours not impacted, other*).

#### Physical and Mental Health

Participants were asked whether they were living with a long-term physical health condition (*yes, no, don’t know, prefer not to say*), and/or a long-term mental health condition (*yes, no, don’t know, prefer not to say*).

The survey also included the validated DASS-21,^6^ which was used to assess symptoms of depression, anxiety, and stress. The DASS-21 is a 21-item measure consisting of 3 subscales (depression, anxiety, and stress), with 7 items per subscale. Participants were asked to read each statement (eg, *I found it hard to wind down*) and indicate how much the statement applied to them over the past week, on a scale from 0 (*did not apply to me at all*) to 3 (*applied to me very much*). Relevant items were combined to give a total score for each of the 3 scales, and severity ratings were given (Normal, Mild, Moderate, Severe, and Extremely Severe) based on this total. The DASS-21 possesses good internal consistency across the subscales and overall scale (α *>* 0.81), and convergent and discriminant validity has been established.^30^

#### Disaster Exposure

Participants were asked whether they had experienced another disaster such as drought, flood, or bushfire in the past 2 years (*yes, no*) and whether they had experienced emotional stress, physical stress, financial stress, damage or loss of property, or other impact because of the disaster(s) they had experienced (and if so, to specify).

### Recoding of Survey Questions

Each participant’s postcode of residence was recoded into the Modified Monash Model categories of 1-7 and subsequently divided into metropolitan (MMM1) and rural, regional or remote (MMM2-7).^31^ Living arrangements were divided into living alone (including *by myself*) and living with others (including, *with my partner, with pre-school-aged children, with school-aged children, with friends or flatmates, with other family, other*). Responses to the pre-existing physical health and mental health condition were limited to yes or no. A new disaster exposure and impact variable, containing 3 categories, was created from the disaster questions: no additional disaster exposure (including participants who answered *no* to having experienced another disaster in the past 2 years), additional disaster exposure with no impact (including participants who answered *yes* to having experienced another disaster in the past 2 years, but did not report an impact from the disaster), and additional disaster exposure with impact (including participants who answered *yes* to having experienced another disaster in the past 2 years and did report an impact from the disaster).

### Statistical Analysis

Categorical variables are presented as *N* and percent (*%*), while continuous variables are presented as mean (*M*) and standard deviation (*SD*). DASS-21 subscale scores were compared to the existing Australian population norms^6^ using an online t-test calculator. Between group comparisons for continuous variables were completed using analysis of variance (ANOVA). Post hoc tests were undertaken for statistically significant results using Tukey’s Honestly Significant Difference test. Separate multiple regression analyses were conducted to explore factors predictive of depression, anxiety, and stress. The independent variables of age, sex, pre-existing chronic health condition, pre-existing mental health condition, and additional disaster exposure with impact were included in the analysis. For the regressions, the variable “sex” was limited to male and female, whereas the disaster exposure with impact variable was recoded into yes or no from the free text responses.

## Results

### Demographics

Demographic characteristics of the participants are depicted in Table 1. Overall, participants were mostly female (80.3%) and ranged in age from 18 to 77 years (*M* = 43.8, *SD* = 12.9). The majority were from non-metropolitan areas (MMM 2-7; 78.1%), lived with other people (87.4%), had not been diagnosed with COVID-19 (98.9%), had not lived with someone diagnosed with COVID-19 (99.1%), worked in the health care and social assistance industry (45.0%), were in paid work (98.0%), worked full time (56.5%), and were either working from home (38.9%) or their normal workplace (35.5%). Over half the participants reported COVID-19 had had no impact on their work hours (67.0%). Approximately one-fifth identified as having a pre-existing chronic disease (21.3%) or mental health condition (21.2%). Over half (76.1%) had had no additional disaster exposure.

**Table 1.** Demographic characteristics of respondents

	Total
**Respondents, n (%)**	596 (100)
**Sex, n (%)**	
Male	106 (19.2)
Female	444 (80.3)
Non-binary	1 (0.2)
Prefer not to say	2 (0.4)
**Age, mean ± SD**	43.8 ± 12.9
**Residence, n (%)**	
Non-metropolitan (MMM2-7)	410 (78.1)
Metropolitan (MMM1)	115 (21.9)
**Living situation**	
Live alone, n (%)	75 (12.6)
Live with others, n (%)	521 (87.4)
**Individual diagnosis of COVID-19**	
Yes, tested and confirmed	0 (0)
Suspected but not tested	3 (0.5)
No	542 (98.9)
Don’t know	3 (0.5)
**COVID-19 diagnosis in another person in the household**	
Yes, tested and confirmed	0 (0)
Suspected but not tested	3 (0.5)
No	542 (99.1)
Don’t know	2 (0.4)
**Pre-existing physical health condition, n (%)**	
Yes	115 (21.3)
No	424 (78.7)
**Pre-existing mental health condition, n (%)**	
Yes	113 (21.2)
No	419 (78.8)
**Industry, n (%)**	
Agriculture, forestry, and fishing	11 (2.0)
Professional, scientific, and technical services	37 (6.7)
Administrative and support services	26 (4.7)
Education and training	131 (23.6)
Health care and social assistance	250 (45.0)
Arts and recreation services	47 (8.5)
Other^a^	88 (15.8)
**Nature of work, n (%)**	
Paid work	553 (98.0)
Volunteer work	11 (2.0)
**Employment type, n (%)**	
Full time	318 (56.5)
Part time	184 (32.7)
Casual	47 (8.3)
Not applicable	2 (0.4)
Other	12 (2.1)
**Work location**	
Home	218 (38.9)
Normal workplace	199 (35.5)
Home and normal workplace combination	102 (18.2)
Not currently working or volunteering	22 (3.9)
Other^b^	19 (3.4)
**Impact of COVID-19 on work hours**	
Temporarily stood down	17 (3.1)
Terminated	8 (1.5)
Hours reduced	40 (7.3)
No impact on hours	365 (67.0)
Other^c^	115 (21.1)
**Disaster exposure type**	
No additional disaster exposure	401 (76.1)
Additional disaster exposure without impact	41 (7.8)
Additional disaster exposure with impact	85 (16.1)

*Notes*:

a Mining, manufacturing, electricity, gas, water and waste services, construction, wholesale trade, retail trade, accommodation and food services, transport, postal and warehousing, information media and telecommunications, financial and insurance services, and public administration and safety.

b Suspended activities, different workplace, changes of work location as restrictions eased.

c Increased hours, increased workload, redeployment, self-employment, leave and/or reduced pay.

### Mental Health

Table 2 shows the proportion of participants who scored across the severity categories of the DASS-21 subscales, as well as the mean and standard deviation for the DASS-21 total subscale scores. Overall, 19.2% of participants were experiencing moderate to extremely severe depression, 13.4% were experiencing moderate to extremely severe anxiety, and 16.8% were experiencing moderate to extremely severe stress. When compared to the existing Australian population norms (pre-pandemic),^6^ DASS-21 scores for depression and stress were significantly higher for participants in this study (6.3 ± 7.0 vs 8.2 ± 9.0, *P* < 0.0001; 10.1 ± 7.9 vs 11.8 ± 9.0, *P* < 0.0001, respectively).

**Table 2.** Depression, anxiety, and stress (DASS-21) severity ratings

	Normal	Mild	Moderate	Severe	Extremely Severe
	**n (%)**	**n (%)**	**n (%)**	**n (%)**	**n (%)**
Depression Subscale	326 (54.7)	70 (11.7)	72 (12.1)	16 (2.7)	26 (4.4)
Anxiety Subscale	380 (63.8)	53 (8.9)	37 (6.2)	19 (3.2)	24 (4.0)
Stress Subscale	361 (60.6)	47 (7.9)	56 (9.4)	32 (5.4)	12 (2.0)

As shown in Table 3, 1-way between-group ANOVAs revealed a significant relationship between disaster exposure type (no additional disaster exposure, additional disaster exposure without impact, additional disaster exposure with impact) and the DASS-21 stress subscale (F (2, 487) = 3.994, *P* = 0.019, η^2^ = 0.016). DASS-21 stress subscale scores were significantly higher for participants who had experienced an additional disaster exposure with impact than for those who had experienced an additional disaster without impact (6.8 ± 4.3 vs 4.4 ± 4.0).

**Table 3.** Mean (standard deviation) depression, anxiety, and stress scores among respondents who had experienced no additional disaster exposure, additional disaster exposure without impact, and additional disaster exposure with impact

	Total Sample	No Additional Disaster Exposure	Additional Disaster Exposure Without Impact	Additional Disaster Exposure With Impact	*P*
Depression Subscale	4.1 ± 4.5	4.0 ± 4.3	3.4 ± 4.5	4.8 ± 5.1	0.227
Anxiety Subscale	2.4 ± 3.2	2.3 ± 3.2	2.3 ± 3.5	2.8 ± 3.2	0.409
Stress Subscale	5.8 ± 4.5	5.8 ± 4.6	4.4 ± 4.0	6.8 ± 4.3	0.019

#### Predictors of Depression, Anxiety, and Stress Severity

Three separate multiple regressions were used to explore factors predictive of DASS-21 depression, anxiety, and stress symptom severity. Sex, age, pre-existing chronic health condition, pre-existing mental health condition, and additional disaster exposure with impact were included as independent variables in each of the analyses. The final multiple regression models are presented in Table 4.

**Table 4.** Predictors of depression, anxiety, and stress severity (DASS-21 subscale scores)

	DASS-21 Depression				DASS-21 Anxiety				DASS-21 Stress			
	B	SE	Beta	*P*	B	SE	Beta	*P*	B	SE	Beta	*P*
**Constant**	2.194	1.775		0.219	1.530	1.233		0.217	5.385	1.599		0.001
**Sex**												
Male	2.127	1.030	0.182	0.041	0.909	0.721	0.113	0.211	0.746	0.940	0.070	0.429
Female	1				1				1			
**Age**	-0.016	0.034	-0.042	0.646	-0.012	0.024	-0.046	0.618	-0.045	0.031	-0.135	0.146
**Chronic health condition**												
**Yes**	0.213	0.968	0.020	0.826	1.195	0.671	0.164	0.078	0.575	0.885	0.060	0.517
**No**	1				1				1			
**Mental health condition**												
Yes	5.060	1.016	0.451	< 0.001	3.013	0.705	0.395	< 0.001	3.289	0.923	0.326	0.001
No	1				1				1			
**Additional disaster exposure with impact**												
Yes	1.475	0.882	0.146	0.097	0.601	0.619	0.087	0.333	2.367	0.790	0.262	0.003
No	1				1				1			

Among participants who had experienced a disaster in the 2 years prior to the survey, higher depression scores were associated with being male (B = 2.127, *P* = 0.041) and having a pre-existing mental health condition (B = 5.060, *P* < 0.001). Higher anxiety scores were associated with having a pre-existing mental health condition (B = 0.3.013, *P* < 0.001). Higher stress scores were associated with pre-existing mental health condition (B = 0.3.289, *P* = 0.001) and having reported an impact from the disaster (B = 2.367, *P* = 0.003). The predictors for depression, anxiety, and stress explained 25.0% (F (5, 105) = 7.008, *P* < 0.001), 22.7% (F (5, 105) = 6.162, *P* < 0.001), and 21.4% (F (5, 109) = 5.939, *P* < .001) of variance, respectively.

## Discussion

This study provides some insight into how the COVID-19 pandemic has impacted the mental health of people living and working in Australia, particularly those who were exposed to a disaster prior to COVID-19. Overall, 19.2%, 13.4%, and 16.8% of participants were experiencing moderate to extremely severe depression, anxiety, and stress symptoms, respectively, as measured by the DASS-21. When compared to the existing Australian population norms^6^ (pre-pandemic), the mean DASS-21 scores for depression and stress were significantly higher for participants in this study. Despite this, the levels found in this study were lower than those found in previous Australian research.^2,32^ For instance, Newby et al.^2^ found that 46.3%, 40.8%, and 38.7% of participants reported moderate to extremely severe symptoms of depression, anxiety, and stress, respectively, whereas Rossell et al.^32^ found that 21-35% of their sample demonstrated moderate to extremely severe depression, anxiety, and stress.

Estimates of the rates of psychological distress are likely to vary based on sample characteristics (eg, reported rates of pre-existing mental health conditions) and contextual factors (eg, survey timing). The proportion of participants reporting a mental health condition in the current study was 21.2%, which is representative of the Australian prevalence rates.^33^ This was not the case for Newby et al.’s study,^2^ where the proportion was closer to 70.0%. Further, the majority of participants in this study were from non-metropolitan areas (78.1%), had not been diagnosed with COVID-19 (98.9%), were in paid work (98.0%), worked full time (56.5%), and reported COVID-19 had not impacted work hours (67.0%). The survey used in this study was also distributed at a time when COVID-19 cases in Australia were relatively under control and restrictions were being eased.^26,27^ Given these characteristics, it is possible this sample was somewhat protected from increased psychological distress during COVID-19; they were likely experiencing fewer COVID-19 cases and less financial stress than other samples. Additionally, these results should be considered in light of the potential mental health benefits of working.^22^ Although unmeasured, other protective factors might be contributing to these findings, including workplace (eg, good-quality supervision^22^), family, or government support.^27,28^ Overall, this highlights the need to carefully interpret research in this area.

The current study also provides insights into the variables associated with higher psychological distress during the COVID-19 pandemic. In particular, and in keeping with previous Australian research,^2,32^ this study found having a pre-existing mental health condition predicted higher severity of depression, anxiety, and stress-related symptoms. This adds to the growing body of evidence highlighting the increased emotional impact of the COVID-19 pandemic on people with a lived experience of mental illness. However, it is also possible that people with a pre-existing mental health condition were experiencing higher levels of psychological distress prior to the COVID-19 pandemic, rather than as a result of the pandemic. It is difficult to differentiate this as no baseline assessment for psychological distress prior to the pandemic was conducted. This is a common limitation in all research related to disasters and mental health, and it will likely be important for future research in this area to adopt a prospective study design to examine prevalence and severity estimates prior to disasters occurring. This study also found being male predicted higher levels of depressive symptoms, but not anxiety or stress. The findings from past Australian research have varied, with 1 study finding being female was predictive of lower depression^2^ and another study finding lower levels of negative emotions were demonstrated by males.^32^ However, international research has consistently found that being female is associated with higher levels of stress, anxiety, and depression.^7,9,10^ Contextual issues between countries and surveys may explain some of this variation.

This study also found that having experienced a disaster in addition to COVID-19, and having experienced an impact from that disaster, was predictive of higher stress symptoms, but not depression or anxiety. This adds to previous research into the mental health impact of COVID-19, which has shown a history of more general stressful events (eg, dismissal, mourning) is predictive of higher levels of depression and anxiety.^10^ Furthermore, it extends this research, by highlighting the need to consider a person’s previous disaster experience when researching mental health outcomes during COVID-19, due to the cumulative effects of repeated disaster on mental health and psychological distress. These cumulative effects have been shown in research unrelated to COVID-19.^17,18,34^ The current study also emphasizes a need to consider how persons are impacted by a disaster, and not just whether they have been exposed to a disaster. Again, this has been suggested by extant literature unrelated to COVID-19.^19,20^ That having experienced a disaster in addition to COVID-19, and having experienced an impact from that disaster, was predictive of higher stress symptoms, but not depression or anxiety, could be related to how the DASS-21 measures these symptoms. The DASS-21 conceives stress as a persistent state of over-arousal reflecting a continuing difficulty in meeting taxing life demands, depression as being related to low self-esteem and anxiety as being related to an increased anticipation of negative events.^6^ It is possible this is reflective of how people emotionally respond to multiple disasters. Alternatively, it is also possible it is too early to see increases in depression and anxiety in our participants; additional life stressors following a disaster are predictive of increased symptomatology over time^15,16^; however, the long-term emotional burden of COVID-19 is still emerging. Further research with larger sample sizes would be needed to properly elucidate this.

### Implications

While it is difficult to ascertain the level of distress participants in this study were experiencing prior to the pandemic, and whether these levels changed as the pandemic began and progressed, there is a need, regardless, to provide an appropriate response to lessen elevated symptoms of psychological distress. The needs of different subgroups of the population will vary widely, and responses will need to be tailored to an individual’s unique combination of risk and protective factors. Responses will likely need to vary in intensity and encompass multiple levels of intervention (eg, societal, community, family, and individual) so as to prevent subclinical symptomatology from worsening over time or developing into psychiatric disorders.^12,35^ Thought will need to be given to the services, organizations, or agencies best placed to deliver such responses, particularly in places where traditional mental health services are limited. In most cases, consideration will also need to be given to including local stakeholders to ensure programs and services are culturally and regionally appropriate.^35^ Research regarding how to provide mental health responses following disasters is still emerging, but it is hoped the larger program of research surrounding this study will address this issue in more detail, with a focus on how organizations can be used to deliver appropriate interventions to improve employee preparedness, response, and recovery.^2,11–13^

### Limitations and Future Research

The results of this study are based on a convenience sample of workers recruited online. Thus, the results are unlikely to generalize to the broader Australian population. Our sample was also small in comparison to other studies in this area, with the majority of participants being female (80.3%), living in a non-metropolitan area (78.1%), and having no additional disaster exposure (76.1%), which might have impacted results. Future research should focus on the mental health impact of having experienced multiple disasters and the implications of this on organizations that may have a role to play in the response, such as workplaces. It would also be helpful to assess in more detail people’s perceived experience of single and multiple disasters, and how this relates to their mental health and well-being.

## Conclusions

There is a growing body of evidence that highlights the increased psychological impact of the COVID-19 pandemic on people with a lived experience of mental illness, as well as those who are experiencing, or have experienced, additional stressors (such as impact from an additional disaster).^2,10,11^ When considered alongside other COVID-19 and mental health research, our study highlights the need to provide an appropriate response to lessen a person’s severity of psychological distress; different subgroups of the population will have varied combinations of risk and protective factors, and responses will need to take this into consideration. Importantly, COVID-19 is not occurring in a vacuum, and as climate-related disasters become more intense, frequent, and longer in duration, increasing attention should be given to the cumulative mental health impact of multiple disasters.
